# Estimation of Tyrosinase-Related Protein 1 (TRP-1) Gene Expression in Human Gingiva and Its Correlation With Gingival Melanin Hyperpigmentation: A Pilot Study

**DOI:** 10.7759/cureus.76843

**Published:** 2025-01-03

**Authors:** Revati S Deshmukh, Rizwan M Sanadi, Meenal Tepan

**Affiliations:** 1 Oral Pathology and Microbiology, Bharati Vidyapeeth Dental College and Hospital, Pune, IND; 2 Periodontology and Oral Implantology, Dr. G.D. Pol Foundation's Y.M.T. Dental College and Hospital, Navi Mumbai, IND; 3 Oral Medicine and Radiology, Bharati Vidyapeeth Dental College and Hospital, Pune, IND

**Keywords:** gingival melanin hyperpigmentation, melanin pigmentation, melanogenesis, tyrosinase, tyrosinase related protein-1

## Abstract

Background

Melanin synthesis in humans relies on the activity of the tyrosinase enzyme, along with tyrosinase-related proteins 1 and 2 (TRP-1 and TRP-2). TRP-1 functions as a 5,6-dihydroxyindole-2-carboxylic acid oxidase and plays a crucial role in activating and stabilizing tyrosinase, as well as in melanosome synthesis. However, TRP-1 gene expression in human gingiva has not been explored. Therefore, this study aimed to estimate TRP-1 gene expression in human gingiva.

Aim

The aim of the study was to estimate TRP-1 gene expression in human gingiva and examine its correlation with the degree of gingival melanin hyperpigmentation.

Materials and methods

Gingival tissue samples were collected from individuals undergoing gingival depigmentation surgery due to concerns about blackish-looking gums. The gingival epithelial tissue was excised under local anesthesia using a surgical scalpel blade. The excised tissue, including a thin layer of underlying connective tissue, was sent to the laboratory for TRP-1 gene expression analysis using the RT-PCR technique.

Results

The levels of TRP-1 gene expression in human gingiva ranged from 0.459 to 0.973. TRP-1 gene expression in the gingival tissues showed a correlation with the degree of gingival melanin hyperpigmentation, with lower expression levels observed at sites with mild to moderate pigmentation and higher levels at sites with severe pigmentation.

Conclusions

TRP-1 gene expression in human gingiva was positively correlated with the degree of gingival melanin hyperpigmentation, suggesting its potential role in the regulation of gingival melanin pigmentation.

## Introduction

Melanin synthesis in humans involves the activity of the tyrosinase enzyme along with tyrosinase-related proteins 1 and 2 (TRP-1 and TRP-2) [1]. Tyrosinase is a critical enzyme that catalyzes the conversion of L-tyrosine to L-3,4-dihydroxyphenylalanine (L-DOPA) [2]. TRP-1 functions as a 5,6-dihydroxyindole-2-carboxylic acid (DHICA) oxidase [1] and plays a role in the activation and stabilization of tyrosinase as well as in melanosome synthesis. TRP-1 has also been suggested to increase the ratio of eumelanin to pheomelanin [3].

TRP-1 is associated with the differentiation of melanocytes and the enzymatic processes involved in pigmentation [4], and it helps protect melanocytes from oxidative stress [5]. While the role of TRP-1 and TRP-2 in the eumelanogenic pathway remains unclear [3], TRP-1 has been shown to catalyze the oxidation of DHICA in mice, though this activity in humans is not yet fully understood [1].

While studies have explored the role of dermal melanocytes in melanin synthesis and their correlation with tyrosinase activity, no study has yet investigated TRP-1 gene expression in human gingiva and its correlation with the degree of gingival melanin hyperpigmentation. Therefore, this study was conducted to estimate TRP-1 gene expression in human gingiva.

## Materials and methods

**Figure 1:**
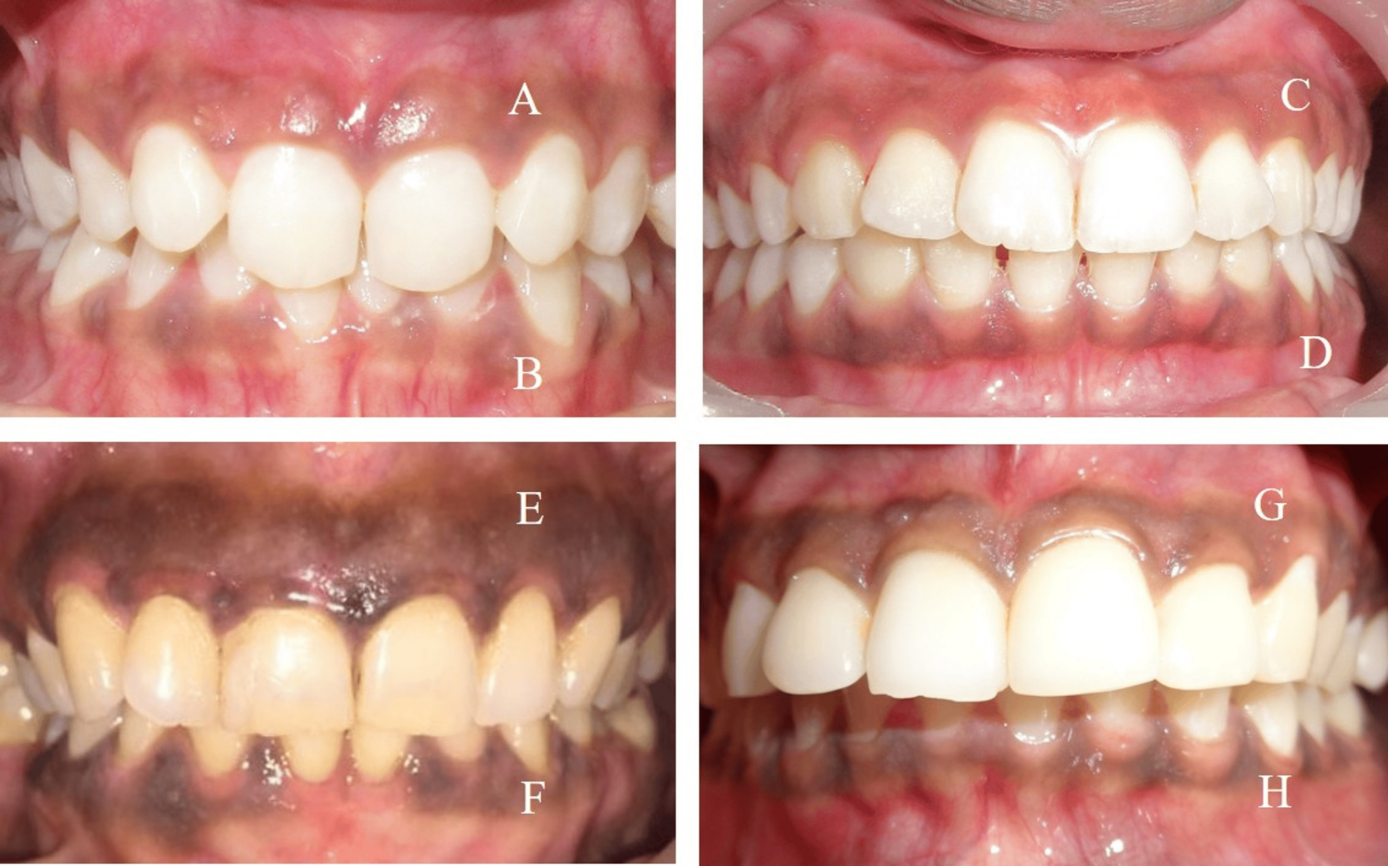
Intraoral photographs of the sample sites exhibiting pigmentation (A) Intraoral photograph showing Site 1 (maxillary gingiva) with moderate pigmentation. (B) Intraoral photograph showing Site 2 (mandibular gingiva) with moderate pigmentation. (C) Intraoral photograph showing Site 3 (maxillary gingiva) with severe pigmentation. (D) Intraoral photograph showing Site 4 (mandibular gingiva) with severe pigmentation. (E) Intraoral photograph showing Site 5 (maxillary gingiva) with severe pigmentation. (F) Intraoral photograph showing Site 6 (mandibular gingiva) with severe pigmentation. (G) Intraoral photograph showing Site 7 (maxillary gingiva) with moderate pigmentation. (H) Intraoral photograph showing Site 8 (mandibular gingiva) with severe pigmentation.

Gingival tissue samples were collected from systemically healthy individuals who underwent gingival depigmentation surgery due to concerns about blackish-looking gums. Gingival melanin hyperpigmentation was graded using the Dummett-Gupta Oral Pigmentation Index (DOPI) [6]. Tissue samples from individuals with a DOPI score of 2 (moderate pigmentation) and score 3 (heavy pigmentation) were included in the study. Figure 1 shows intraoral photographs of sample sites with pigmentation.

The gingival epithelial tissue, along with a thin layer of underlying connective tissue, was excised under local anesthesia using a surgical scalpel blade. The excised tissue was then sent to the laboratory in Eppendorf tubes containing phosphate-buffered saline (PBS) for TRP-1 gene expression estimation.

The procedural steps for RT-PCR were as follows: (1) The tissues were cut into smaller pieces and digested with a collagenase/dispase enzymatic cocktail at 37°C. (2) RNA was isolated using the triazole method, as recommended by Thermo Fisher Scientific (Waltham, Massachusetts, USA), following the manufacturer’s guidelines. (3) The SuperScript® III Cells Direct cDNA Synthesis Kit was used to isolate cDNA from the cells. (4) RT-PCR analysis was performed using the Applied Biosystems QuantStudio 5 (QS5) Real-Time PCR system. (5) The cycling conditions were 50°C for two minutes, 95°C for 10 minutes, followed by 40 cycles of 95°C for 15 seconds and 60°C for one minute. (6) TRP-1 gene expression levels were estimated.

The information contained in DNA is transcribed into mRNA by the transcription machinery. The level of gene expression can be quantified by measuring the amount of mRNA produced, providing a quantitative analysis of its presence.

## Results

Table 1 presents the DOPI scores and the corresponding levels of TRP-1 gene expression in human gingiva. The levels of TRP-1 gene expression in the gingival tissues ranged from 0.459 to 0.973. TRP-1 gene expression was found to correlate with the degree of gingival melanin hyperpigmentation, with lower expression levels observed at sites with moderate pigmentation and higher levels at sites with severe pigmentation.

**Table 1:** DOPI score and TRP-1 gene expression DOPI score 2 indicates moderate pigmentation, and DOPI score 3 indicates severe pigmentation. DOPI, Dummett-Gupta Oral Pigmentation Index; TRP-1, tyrosinase-related protein 1

Site (x-axis)	DOPI score	TRP-1 gene expression (y-axis)
Site 1	2	0.569
Site 2	2	0.459
Site 3	3	0.728
Site 4	3	0.693
Site 5	3	0.973
Site 6	3	0.684
Site 7	2	0.618
Site 8	3	0.668

## Discussion

Melanogenesis is the process of melanin synthesis that occurs in the melanocytes located in the basal layer of the epithelium. Melanin absorbs ultraviolet radiation and protects the skin from its harmful effects. The synthesis of melanin is regulated by various intrinsic and extrinsic factors [7].

Tyrosinase is an enzyme exclusively produced by melanocytes that plays a key role in melanin synthesis. It stabilizes the tyrosinase protein while altering its catalytic function [8]. The gene also encodes TRP-1, an intermediate enzyme involved in melanin synthesis, which is expressed in melanocytes, the cells responsible for melanin production.

TRP-1 is a 75 kDa transmembrane glycoprotein synthesized in the endoplasmic reticulum and transported by the Golgi apparatus to the melanosomes. Its mature form was initially called gp75. The gene for TRP-1 is located on chromosome 9 (9p23) [9].

TRP-1 contains four conserved regions: the signal peptide (residues 1-24), the intramelanosomal domain (25-477), which is further subdivided into the Cys-rich (25-126) and tyrosinase-like (127-477) subdomains, the transmembrane α-helix (478-501), and the cytoplasmic domain (502-537) [1].

Along with tyrosinase, TRP-1 helps prevent the premature death of melanocytes by reducing tyrosinase-mediated cytotoxicity [10]. TRP-1 has also been used as a marker for melanocyte differentiation due to its role in promoting melanogenesis [11].

The degree of melanin pigmentation in the skin has been correlated with tyrosinase enzyme levels [12]. While the role of dermal melanocytes has been extensively studied, the role of oral mucosal melanocytes in health and disease remains unclear [13,14].

A positive correlation between tyrosinase gene expression and gingival melanin pigmentation has been reported [15]. Individuals with severe melanin pigmentation tend to exhibit higher levels of tyrosinase expression, suggesting that higher levels of pigmentation are associated with increased tyrosinase, mRNA, and protein levels.

The tyrosinase gene provides instructions for synthesizing the tyrosinase enzyme, which converts the amino acid tyrosine into melanin. Variations in the tyrosinase gene can affect the amount and activity of the enzyme, leading to changes in melanin synthesis.

It was observed that tissue samples from sites with severe melanin pigmentation had higher levels of tyrosinase gene expression compared to those from sites with moderate pigmentation. This suggests a positive correlation between the degree of gingival melanin hyperpigmentation and TRP-1 gene expression.

Mutations in the tyrosinase or TRP-1 genes can result in oculocutaneous albinism, a group of autosomal recessive disorders characterized by reduced melanin production in the skin, hair, and eyes.

The variations and significance of the tyrosinase gene in regulating pigmentation play a crucial role in overall pigmentation of the skin and mucosa. However, no studies have yet assessed TRP-1 levels in human gingiva. Therefore, the present study was conducted to evaluate TRP-1 levels in human gingiva and correlate them with the degree of gingival melanin hyperpigmentation. The results of this study suggest a positive correlation between TRP-1 levels and the degree of gingival melanin hyperpigmentation.

Limitations

As mentioned earlier, there has been no study conducted to estimate TRP-1 levels in human gingiva. Therefore, we conducted this pilot study on a small sample size, which serves as a limitation of the study.

## Conclusions

TRP-1 gene expression plays a significant role in gingival hyperpigmentation. Further exploration of its downregulation is needed to better understand the clinically reduced activity of TRP-1 gene expression. Based on the findings of this study, it can be concluded that there is a positive correlation between TRP-1 gene expression levels and the degree of gingival melanin hyperpigmentation in human gingiva. This original research holds translational value and has the potential to advance patient diagnosis, treatment, and prognosis.
